# PTEN loss and level of HER2 amplification is associated with trastuzumab resistance and prognosis in HER2-positive gastric cancer

**DOI:** 10.18632/oncotarget.23054

**Published:** 2017-12-09

**Authors:** Chan Kim, Choong-Kun Lee, Hong Jae Chon, Joo Hoon Kim, Hyung Soon Park, Su Jin Heo, Hyun Jeong Kim, Tae Soo Kim, Woo Sun Kwon, Hyun Cheol Chung, Sun Young Rha

**Affiliations:** ^1^ Medical Oncology, CHA Bundang Medical Center, CHA University, Seongnam, Korea; ^2^ Yonsei Cancer Center, Yonsei University College of Medicine, Seoul, Korea; ^3^ Song Dang Institute for Cancer Research, Seoul, Korea; ^4^ Brain Korea 21 PLUS Project for Medical Science, Yonsei University College of Medicine, Seoul, Korea

**Keywords:** trastuzumab, resistance, gastric cancer, HER2, PTEN

## Abstract

**Background:**

Trastuzumab is an active agent against human epidermal growth factor receptor 2 (HER2)-positive gastric cancer (GC). This study aimed to characterize resistance to trastuzumab-based front-line chemotherapy in HER2+ GC patients and to establish factors predictive of this resistance.

**Results:**

Among 129 HER2+ GC patients, 25% displayed rapid disease progression within 4 months from initiation of therapy. These patients showed a higher rate of signet ring cell histology, bone metastasis, poor performance status, frequent loss of PTEN expression, and low HER2 amplification index compared with patients who were progression-free for at least 4 months. In contrast, there was no significant difference in the frequency of the PIK3R1 variant. Multivariate analyses confirmed two independent molecular predictors for trastuzumab resistance: loss of PTEN expression and low HER2 amplification index (<5). Patients with one or both molecular predictors at diagnosis exhibited worse progression-free and overall survival compared to those without risk factors (*p* < 0.001 and *p* = 0.001, respectively).

**Conclusion:**

In HER2+ GC patients, loss of PTEN expression and low HER2 AI correlated with resistance to trastuzumab-based therapy and dismal prognosis. Since patients harboring these molecular predictors are unlikely to respond to trastuzumab-based therapy, other novel therapeutic targets needed to be considered.

**Methods:**

HER2+ GC patients who were treated with trastuzumab in combination with either 5-fluorouracil/cisplatin or capecitabine/cisplatin were enrolled. Clinicopathologic features and molecular alterations of HER2, phosphoinositide 3-kinase regulatory subunit 1 (PIK3R1), and phosphatase and tensin homolog (PTEN) were correlated with treatment outcome. Factors predictive of resistance were also explored.

## INTRODUCTION

Trastuzumab (Herceptin, Roche) is a potent anti-HER2 humanized monoclonal antibody directed against HER2+ gastric cancer (GC) [1, 2]. The pivotal ToGA (Trastuzumab for Gastric Cancer) trial showed that addition of trastuzumab to conventional chemotherapy significantly prolongs overall survival (OS) and progression-free survival (PFS) in HER2+ GC compared with chemotherapy alone [3]. Trastuzumab-based regimen is now being considered as a standard front-line treatment for patients with HER2+ GC, and the median OS of these patients has risen to >12 months [3–5]. However, a proportion of HER2+ GC patients do not respond to trastuzumab-containing front-line chemotherapy *ab initio*, progressing rapidly within 3–4 months, and displaying dismal prognosis despite treatment [3, 6]. These patients with refractory malignancies seem to have primary (*de novo*) resistance and currently, detailed characterization of these patients and availability of surrogate marker to predict primary resistance remain unknown.

Activation of the PI3K signaling pathway is common in various human malignancies. Moreover, the deregulation of PI3K subunits and PTEN are well-known to be involved during carcinogenesis [7–9]. Specifically, a weakened response to trastuzumab therapy was often seen in HER2+ breast cancer containing activating mutations of the PIK3CA gene and deletions of PTEN [10, 11]. It has also been found that PTEN is not only a tumor suppressor, but also plays a critical role in trastuzumab susceptibility of HER2-amplified breast cancer cell lines [12]. Recently, PIK3R1, the regulatory subunit of PI3K, was also known to be involved in the activation of PI3K pathway and cancer progression [7, 13]. Genetic aberrations of PIK3R1 was reported in various tumors [13–16] and, intriguingly, tumors lacking PIK3CA mutations tended to have frequent mutations in PIK3R1 [15]. PIK3R1 mutant protein is known to bind and stabilize PTEN, thereby suppressing PTEN degradation in tumor cell [13]. In a previous study [17], we were unable to confirm the predictive role of PIK3CA in HER2+ GC; therefore, here we aimed to identify the role of other regulators of the PI3K signaling pathway in HER2+ GC: PTEN and PIK3R1.

In this study, we aimed to characterize HER2+ GC patients with resistance to trastuzumab-based therapy by focusing on the PI3K pathway, in order to establish molecular predictive factors for primary resistance.

## RESULTS

### Baseline patient characteristics

For 10 years, a total of 129 HER2+ GC patients were enrolled and treated with either trastuzumab/XP or trastuzumab/FP as a first-line chemotherapy. The clinicopathologic and molecular characteristics of HER2+ GC patients are summarized in Table 1. The median age at diagnosis was 59 years (range: 30–84) and the male-to-female ratio was 2.9:1. Two-thirds of patients initially presented with distant metastases, whereas cancer recurred after previous curative gastrectomy in the remaining third. The most frequent metastatic site was the liver (41.1%), followed by peritoneum (34.9%), lung (12.4%) and bone (11.6%).

**Table 1:** Clinicopathologic features of HER2+ gastric cancer

	All patients (*n =* 129)	Trastuzumab-resistant patient (*n =* 32)	Trastuzumab-sensitive patients (*n =* 97)	*P*-value
Age at diagnosis	59 (30-84)	60 (30-82)	58 (32-84)	0.623
Sex				0.931
Male	96 (74.4%)	24 (75.0%)	72 (74.2%)	
Female	33 (25.6%)	8 (25.0%)	25 (25.8%)	
Histology				0.031
Adeno, WD	7 (5.4%)	0 (0%)	7 (7.2%)	
Adeno, MD	76 (58.9%)	16 (50.0%)	60 (61.9%)	
Adeno, PD	33 (25.6%)	9 (28.1%)	24 (24.7%)	
SRC carcinoma	13 (10.1%)	7 (21.9%)	6 (6.2%)	
Previous surgery				0.491
Yes	42 (32.6%)	12 (37.5%)	30 (30.9%)	
No	87 (67.4%)	20 (62.5%)	67 (69.1%)	
Location of tumor				0.420
Proximal	31 (24.0%)	6 (18.8%)	25 (25.8%)	
Distal	98 (76.0%)	26 (81.3%)	72 (74.2%)	
Peritoneal metastasis				0.225
Yes	45 (34.9%)	14 (43.8%)	31 (32.0%)	
No	84 (65.1%)	18 (56.3%)	66 (68.0%)	
Liver metastasis				0.951
Yes	53 (41.1%)	13 (40.6%)	40 (41.2%)	
No	76 (58.9%)	19 (59.4%)	57 (58.8%)	
Bone metastasis				0.025
Yes	15 (11.6%)	8 (25.0%)	7 (7.2%)	
No	104 (80.6%)	22 (68.8%)	82 (84.5%)	
N/A	10 (7.8%)	2 (6.3%)	8 (8.2%)	
Lung metastasis				0.985
Yes	16 (12.4%)	4 (12.5%)	12 (12.4%)	
No	113 (87.6%)	28 (87.5%)	85 (87.6%)	
Performance status				0.001
ECOG 0	72 (55.8%)	8 (25.0%)	64 (66.0%)	
ECOG 1	42 (32.6%)	18 (56.3%)	24 (24.7%)	
ECOG ≥2	15 (11.7%)	6 (18.7%)	9 (9.2%)	
Regimen				0.896
Herceptin-FP	17 (13.2%)	4 (12.5%)	13 (13.4%)	
Herceptin-XP	112 (86.8%)	28 (87.5%)	84 (86.6%)	
HER2 IHC				0.475
2+	31 (24.0%)	10 (31.3%)	21 (21.6%)	
3+	98 (76.0%)	22 (68.8%)	76 (78.4%)	
HER2 amplification index	7.0 ± 5.9	7.7 ± 6.3	4.1 ± 1.4	0.005
PTEN expression				0.017
Loss	11 (16.4%)	5 (38.5%)	6 (11.1%)	
Intact	56 (83.6%)	8 (61.5%)	48 (88.9%)	
PIK3R1 genotype				0.460
Variant	16 (23.2%)	5 (31.3%)	12 (22.2%)	
Wildtype	53 (76.8%)	11 (68.8%)	42 (77.8%)	

### HER2, PTEN, and PIK3R1 status and their association with patient characteristics

HER2 expression in IHC staining was 3+ in 98 (76.0%) patients and 2+ in the remaining 31 (24.0%) patients. The mean amplification index (AI) of HER2 expression determined by FISH analysis was 7.0. The median follow-up duration was 23.8 months (range 0.9–61.8). We observed intact PTEN expression in 83.6% of GC tissues and loss of PTEN expression in 16.4% of GC tissues. The PIK3R1 wildtype allele (GG) was identified in 76.8% of patients, while the remaining 23.2% had a variant allele (GA or AA).

In 129 trastuzumab-treated patients, median PFS was 7.4 months and median OS was 16.0 months. Because there is no consensus on the criteria for dividing GC patients into trastuzumab-sensitive or -resistant, the lower quartile value of PFS (4.0 months) was used as a cut-off value to distinguish between patients who benefitted clinically from trastuzumab and those who did not. As a result, 32 patients (25%) with PFS shorter than 4 months were classified as resistant and 97 patients (75%) with more than 4 months, as sensitive.

The association between clinicopathological and molecular features in trastuzumab-resistant patients are also summarized in Table 1. In HER2+ GC patients with primary resistance, increased signet ring cell (SRC) histology (21.9% vs. 6.2%, *p =* 0.031) and a higher frequency of initial bone metastasis (25.0% vs. 7.2%, *p <* 0.025) was observed along with poor performance status (ECOG ≥2: 18.7% vs. 9.2%, *p =* 0.001). Notably, the existence of initial bone metastasis was highly associated with poor performance status (*p =* 0.002), suggesting that bone metastasis in GC severely impairs the patient’s activities of daily living. The average AI of HER2 in FISH analysis was significantly lower in patients with primary resistance compared with sensitive patients (7.7 ± 6.3 vs. 4.1 ± 1.4, *p =* 0.005). Moreover, loss of PTEN expression was more common in trastuzumab-resistant HER2+ GC patients than in those who were sensitive to treatment (38.5% vs. 11.1%, *p =* 0.017). However, there was no significant difference in the frequency of the PIK3R1 variant allele between patients with and without trastuzumab-resistance.

### HER2 and PTEN status and clinical response to trastuzumab-based therapy

Next, we analyzed clinical outcomes according to the molecular characteristics of HER2+ GC. In terms of tumor response to first-line therapy (Table 2), objective response rate was significantly increased in patients with HER2 AI ≥5 compared to those with AI <5 (58.8% vs. 21.4%, *p =* 0.002). At the same time, objective response rate tended to decrease in patients with a loss of PTEN compared to those with intact PTEN expression (64.3% vs. 36.4%, *p =* 0.220).

**Table 2:** Clinical response to trastuzumab-based first-line chemotherapy

	HER2 AI		PTEN expression	
	≥5	<5	Intact	Loss
Best response				
CR, PR	47 (58.8%)	6 (21.4%)	36 (64.3%)	4 (36.4%)
SD	26 (32.5%)	15 (53.6%)	15 (26.8%)	5 (45.5%)
PD	7 (8.8%)	7 (25.0%)	5 (8.9%)	2 (18.2%)
*P*-value	0.002		0.220	

### Low HER2 amplification index and loss of PTEN expression predict resistance to trastuzumab-based first-line chemotherapy

**Figure 1:**
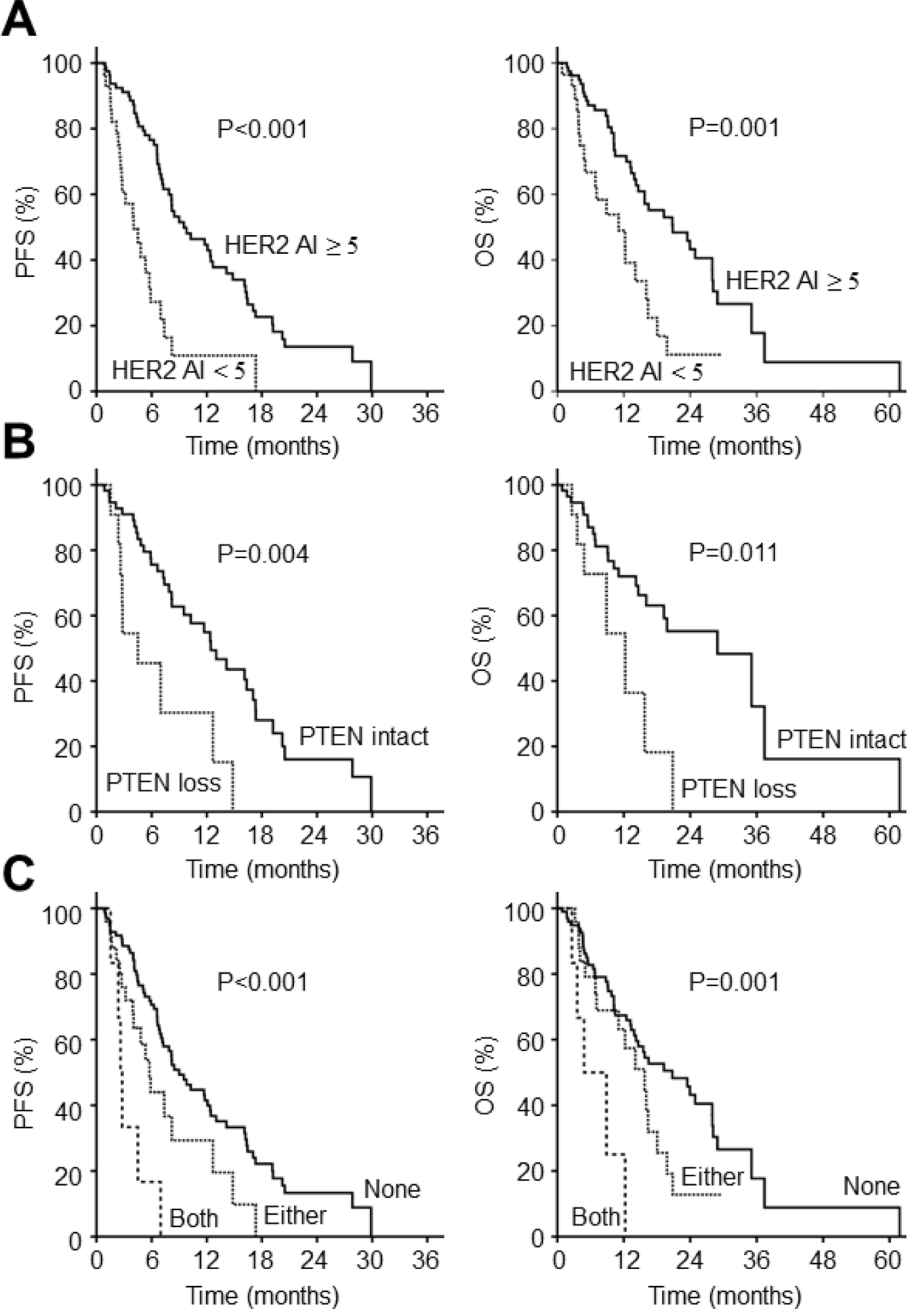
Survival outcomes according to molecular biomarkers in HER2+ gastric cancer (**A**) PFS and OS according to the level of HER2 amplification (**B**) PFS and OS according to the presence or absence of PTEN expression (**C**) PFS and OS according to the number of molecular biomarker PFS, progression-free survival; OS, overall survival.

To identify the factors predictive of response to trastuzumab-based first-line therapy, we performed univariate analyses with various clinicopathologic and molecular characteristics of first-line PFS and OS (Table 3). In univariate analysis, SRC histology, initial presence of bone metastasis, poor performance status, HER2 AI <5, and loss of PTEN expression were predictive for shorter PFS in HER2+ GC patients when treated with Herceptin. Next, to verify independent predictive factors for primary resistance, we performed multivariate analyses for variables found to be statistically significant in univariate analyses. Multivariate Cox regression analysis revealed two independent predictors for primary resistance against trastuzumab-based regimen: low HER2 AI (<5) and loss of PTEN expression. In patients with HER2 AI <5, median PFS and OS of first-line therapy was lower compared to those with HER2 AI ≥ 5 (PFS: 4.0 vs. 9.5 months, *p <* 0.001; OS: 11.1 vs. 20.8 months, *p =* 0.001) (Figure 1A). Moreover, in patients with a loss of PTEN expression, median PFS and OS of first-line trastuzumab-based treatment was shortened compared to those with intact PTEN expression (PFS: 4.5 vs. 12.4 months, *p =* 0.004; OS: 12.3 vs. 28.9 months, *p =* 0.011) (Figure 1B).

**Table 3:** Univariate and multivariate analysis for PFS and OS

	Univariate		Multivariate	
	Median survival (months)	*P*-value	HR (95% CI)	*P*-value
**PFS**				
Histology (SRC vs. non-SRC)	5.1 vs. 8.2	0.103	1.27 (0.46–3.49)	0.647
Bone metastasis (Yes vs. No)	2.8 vs. 8.2	0.001	1.57 (0.57–4.33)	0.383
ECOG Performance (≥1 vs. 0)	5.1 vs. 12.1	<0.001	1.14 (0.52–2.49)	0.742
HER2 AI (<5vs. ≥5)	4.0 vs. 9.5	<0.001	3.26 (1.38–7.73)	0.007
PTEN expression (loss vs. intact)	4.5 vs. 12.4	0.004	3.01 (1.22–7.46)	0.017
**OS**				
Histology (SRC vs. non-SRC)	6.5 vs. 16.4	0.010	1.58 (0.43–5.76)	0.491
Bone metastasis (Yes vs. No)	14.6 vs. 16.9	0.009	2.21 (0.70–7.02)	0.179
ECOG Performance (≥1 vs. 0)	9.8 vs. 23.5	<0.001	1.01 (0.37–2.74)	0.987
HER2 AI (<5vs. ≥5)	11.1 vs. 20.8	0.001	3.23 (1.23–8.49)	0.018
PTEN expression (loss vs. intact)	12.3 vs. 28.9	0.011	2.88 (1.02–8.10)	0.045

Next, we analyzed PFS and OS according to the number of these risk factors that patients possessed: 0 vs. 1 vs. 2. (Figure 1C). The most favorable survival outcome was seen in patients with no risk factors: median PFS was 9.0 months (95% CI: 6.8–11.2) and OS 20.8 months (95% CI: 12.8–28.7). The prognosis of patients possessing 1 risk factor was intermediate with a median PFS of 5.8 months (95% CI: 6.8–11.2) and OS of 15.8 months (95% CI: 9.4–22.1). However, the patients with both risk factors had the worst prognosis with a median PFS of 2.6 months (95% CI: 2.1–3.1) and OS of 4.8 months (95% CI: 0–9.9). Based on these findings, we could confirm that these two molecular factors are the main predictors of primary resistance to trastuzumab in HER2+ GC patients.

## DISCUSSION

Trastuzumab is an active agent used in combination with chemotherapy against HER2+ metastatic GC [1]. Since its approval by the FDA in 2010, it is widely used in the treatment of metastatic HER2+ GC. In Eastern countries, the majority of patients treated with trastuzumab-based chemotherapy presented with PFS > 6 months and OS > 12 months [17, 18]. However, some HER2+ GC patients exhibited primary resistance to trastuzumab-based regimen. These patients progressed rapidly within a few months of initiation of therapy, and eventually displayed dismal prognosis [6].

To date, HER2+ GC patients with primary resistance to trastuzumab have not been thoroughly characterized, and only a few studies on the predictive biomarkers for trastuzumab resistance in GC have been reported. In the post-hoc analysis of the ToGA trial, a link was reported between the baseline HER2 expression level and trastuzumab response [3, 18]. In addition, Gomez-Martin *et al.* recently suggested that the level of HER2 amplification predicts treatment response and overall survival in GC [19]. In most recent study, Deguchi *et al.* revealed that the knockdown of PTEN induces trastuzumab resistance by Akt activation in preclinical model of GC [20]. They also reported that there is no clinical response in PTEN-deficient GC patients compared to response rate of 50% in PTEN-positive GC patients. However, since the number of HER2+ patients in this study was very small (*n =* 23), the results should be interpreted with care. Moreover, there are reports on the relationship between PTEN expression and response to the trastuzumab-containing therapy in HER2+ breast cancer [21]. In summary, comprehensive studies on the potential biomarkers of HER2+ GC, whether they be clinical or molecular, are still lacking and need to be pursued.

In the present study, we found that trastuzumab-resistant HER2+ GC patients usually presented with SRC histology, initial bone metastasis, poor performance status (ECOG ≥ 2), low HER2 AI in FISH and a loss of PTEN expression at diagnosis. Although we also examined the role of PIK3R1, the regulatory subunit of PI3K, we were unable to determine its role in the treatment of HER2+ GC. Moreover, following multivariate Cox regression analysis, we were able to identify two independent molecular predictors for resistance to first-line trastuzumab-containing chemotherapy: low HER2 AI (<5) and loss of PTEN expression. These two risk factors are also independent prognostic factors for OS. Patients possessing either of these risk factors exhibited primary resistance to trastuzumab and their prognosis is dismal, suggesting that this subgroup would benefit from other therapeutic targets. Because loss of PTEN expression is associated with the activation of PI3K signaling and mitogen-activated protein kinase (MAPK) pathways [22], pharmacologic inhibition of these pathways could provide a reasonable strategy to overcome trastuzumab resistance in HER2+ GC.

In this study, patients with SRC histology responded poorly to trastuzumab-based regimen. Previous research has shown that epithelial-to-mesenchymal transition (EMT) confers primary resistance to trastuzumab in tumor cells [23]. In addition, the loss of E-cadherin in gastric mucosal epithelial cells is a critical carcinogenic event that initiates gastric SRC carcinoma in humans and mice [24]. Therefore, primary resistance in SRC carcinoma may be the result of augmented EMT in E-cadherin-deficient SRC. More preclinical evidence is required to prove this inference.

The findings of this study may be limited by its retrospective nature. However, we believe that these shortcomings can be offset to a certain extent by the fact that more than half of the patients in this study formed part of well-controlled, multi-institutional prospective clinical trials such as the ToGA trial, and that the remaining patients received standardized clinical treatment at a single tertiary cancer center. To validate the findings of the present study and further characterize the biology of HER2+ GC, we are now planning to establish the multicenter prospective cohort for HER2+ GC patients in Korea.

In conclusion, loss of PTEN expression and low HER2 AI correlated with resistance to trastuzumab-based first-line therapy and dismal prognosis in HER2+ GC. Since patients harboring these molecular alterations are unlikely to respond to conventional trastuzumab-based therapy, other novel therapeutics that will benefit this subset need to be pursued.

## MATERIALS AND METHODS

### Patient selection

Patients with metastatic GC were enrolled in Yonsei Cancer Center, Severance Hospital, Seoul, Korea between December 2005 and August 2015. Patients were followed up until December 2016. Patient eligibility criteria were as follows: 1) metastatic GC patients with HER2 positivity defined by either HER2 3+ in immunohistochemical (IHC) staining, or HER2 2+ in IHC staining and HER2 amplification on fluorescence *in situ* hybridization (FISH) (HER2:CEP17 ratio ≥2); 2) chemotherapy-naive patients with the exception of adjuvant chemotherapy 6 months prior to enrollment; 3) patients who have been treated with palliative trastuzumab in combination with either 5-fluorouracil and cisplatin (FP) or capecitabine and cisplatin (XP). Clinicopathologic parameters were reviewed from the information of the electronic medical record system as previously described [25].

HER2 status in surgical or biopsy specimen were analyzed and determined by experienced pathologists in Severance Hospital, Seoul, Korea, using the HercepTest Kit™ (DAKO, Denmark) for IHC staining and Vysis™ HER2/CEP FISH probe kit (Abbott, USA) for FISH analysis according to the manufacturers’ instructions. This study was approved by the Institutional Review Board in Severance Hospital, Seoul, Korea (IRB approval number: 4-2014-1076).

### Evaluation of PTEN expression in GC tissue

Formalin-fixed paraffin-embedded sections of tumor tissue were deparaffinized with xylene and hydrated with graded alcohol. Antigen retrieval was performed with a retrieval solution (DAKO, USA) using the pressure-cooking method, and the activity of endogenous peroxidase was blocked by a 1:40 mixture of hydrogen peroxide and methanol. The primary PTEN antibody (DAKO) incubation was performed at room temperature for 1 h in an antibody solution diluted to 1:100. All sections were incubated at room temperature for 30 min in the Real EnVision™ HRP Rabbit/Mouse (DAKO, USA) detection system, which functions as the secondary antibody. PTEN expression was indicated using a chromogen and counterstaining was performed with hematoxylin. PTEN expression was quantified by the H-score based on the intensity of cell staining and percentage of the stained cells [17]. Intensity was scored as 0: none, 1: weak, 2: moderate, or 3: strong. The H-score was calculated as follows: H-score = (%1 + cells × 1) + (%2 + cells × 2) + (%3 + cells × 3). An H-score of ≤ 10 was used as the cutoff point to define loss of PTEN expression based on a previous study.

### PIK3R1 variant analysis

We used a pyrosequencing assay to detect variant alleles of PIK3R1. The primer was forward 5′ (biotin) - CACCAAAACCTACTACTGTAGCCAA-3′ and reverse 5′ - GAGATATCTCCCCAGTACCATTCA-3′. Amplicon length was 85 bp and sequence to analyze was 5′–AGGA C/T ATATT-3′. Each PCR mix contained the forward and reverse primers, dNTP mix, MgCl_2_, PCR buffer, AmpliTaq Gold, and 100 ng of sample genomic DNA. PCR mixtures were denatured for 10 min at 95°C and then thermal-cycled for 30 s at 95°C, 30 s at 55°C, and 30 s at 72°C, repeating the cycle 40 times. A final extension step at 72°C for 5 min completed the program. PCR products were analyzed by agarose gel electrophoresis and sequenced using the PyroMark Q24 (QIAGEN) system according to the manufacturer’s instructions.

### Statistical analysis

The correlation between clinicopathologic variables was compared using chi-square test and independent sample *t*-test. Multivariate analysis was done by logistic regression to identify the independent predictive factors for primary resistance to trastuzumab. OS was defined as the time interval from the date of initiation of trastuzumab-based chemotherapy to the date of death or last follow-up. PFS was defined as the time between initiation of therapy and the date of documented disease progression or death. All patients underwent response evaluation with regular CT scan at 6- to 8-week intervals for accurate PFS measurement. OS and PFS were compared using Kaplan-Meier survival analyses with log-rank tests. The accepted level of statistical significance was *p* < 0.05. All statistical analyses were carried out with SPSS 12.0 (SPSS, Inc.).
